# From intention to action: student and faculty perspectives on private and public campus pro-environmental behavior

**DOI:** 10.3389/fpsyg.2026.1934478

**Published:** 2026-09-07

**Authors:** Xiangen Chen, Jinyan Huang, Xisheng Huang

**Affiliations:** 1 Chongqing University, Chongqing, China; 2 Jiangsu University, Zhenjiang, China

**Keywords:** campus sustainability, collective efficacy, institutional reliability, institutionally situated agency, living laboratories, private and public pro-environmental behavior

## Abstract

Universities increasingly promote sustainability, yet environmental intentions do not consistently become private or public action. This qualitative study examined how university students and faculty in eastern China understood the personal, social, pedagogical, and institutional conditions shaping campus pro-environmental behavior. Four focus groups with 20 undergraduate and graduate students from arts and science disciplines and 10 semi-structured faculty interviews were analyzed using a hybrid deductive-inductive framework approach informed by an institutionally situated extended Theory of Planned Behavior. Six interrelated themes showed that intention was necessary but conditional on situated agency, habit, workload, convenience, health, safety, and institutional support. Perceived behavioral control was analytically distinguished from institutional reliability: the former concerned participants’ appraisals of capability and opportunity, whereas the latter concerned whether facilities, procedures, information, and response systems actually worked. Public participation additionally required collective efficacy grounded in student voice, visible outcomes, organizational continuity, and institutional responsiveness. Environmental knowledge became actionable when local, practical, and linked to meaningful opportunities. Co-designed living laboratories emerged as a participant-generated proposal, not an intervention conducted in the study. The findings explain how curriculum, operations, and governance jointly condition private and public environmental action.

## Introduction

Higher education institutions are expected to advance sustainable development through teaching, research, governance, campus operations, and community engagement (Fang and O’Toole, 2023). Universities function simultaneously as educational institutions, workplaces, residential communities, and organizational systems. Students develop environmental knowledge and values while making repeated decisions about resource use, waste, transport, consumption, and collective participation. Campuses can therefore serve as living laboratories in which sustainability is learned, practiced, and institutionalized (Collado et al., 2022; Findler et al., 2019; United Nations Educational, Scientific and Cultural Organization, 2020).

Campus pro-environmental behavior includes private and public forms. Private behavior comprises routine personal practices such as conserving electricity and water, recycling, reducing waste, and limiting disposable consumption. Public behavior comprises visible and collective actions such as joining campaigns, encouraging peers, sharing information, participating in student organizations, and supporting university sustainability policies (Markle, 2013; Mateer et al., 2022; Stern, 2000). The research object in this study is the translation of environmental intention into these two forms of campus action. The central question is how students’ agency, social relations, faculty practice, and institutional conditions enable or constrain that translation. The scope is campus-based practice and participation across 10 universities in eastern China; the study does not assess off-campus activism, causal effects, or measured environmental outcomes.

Favorable attitudes, however, do not always produce consistent action. This intention–behavior gap may arise from inconvenience, competing priorities, weak infrastructure, unsupportive norms, limited feedback, or doubts that individual and collective actions will matter (Bamberg and Möser, 2007; Kollmuss and Agyeman, 2002). Students may intend to recycle but encounter inadequate facilities, wish to conserve energy but lack control over dormitory systems, or avoid campaigns because of workload, poor communication, or distrust of symbolic initiatives. Environmental action therefore reflects an interaction between personal motivation and institutional conditions.

The Theory of Planned Behavior (TPB) provides the primary theoretical perspective (Armitage and Conner, 2001; Yuriev et al., 2020). It proposes that behavior is proximally shaped by intention and perceived behavioral control, while intention is influenced by attitudes, subjective norms, and perceived behavioral control (Ajzen, 1991, 2011, 2020). In this study, agency is understood as the capacity to form and enact an intention within perceived and actual opportunities, rather than as unconstrained individual choice. This interpretation is important on campuses because universities structure many of the relevant defaults, facilities, rules, costs, and participation channels.

The framework extends the TPB in a delimited way. Environmental knowledge and connectedness to nature are treated as distal influences that can shape behavioral attitudes and perceived control; personal norms add moral obligation; and collective efficacy is especially relevant to public action (Bandura, 2000; Frick et al., 2004; Mayer and Frantz, 2004; Schwartz, 1977). Institutional reliability is not introduced as another psychological trait. It denotes the experienced quality of the material and organizational context that informs perceived control and the likelihood that intention can become behavior. Planned change literature complements, rather than replaces, the TPB by explaining how aligned changes in curriculum, operations, leadership, and feedback can reduce barriers to action (Bookbinder et al., 2024; Rieg et al., 2021).

This institutionally situated extended TPB specifies the conceptual sequence examined qualitatively: knowledge, nature connection, and moral considerations can inform attitudes and norms; attitudes, norms, and perceived control shape intention; and intention is translated into private or public action under actual institutional conditions. Collective efficacy adds a group-level mechanism for public behavior. The framework therefore guided attention to recognizable constructs while leaving open how participants defined control, trust, responsibility, and effective participation in everyday university life.

A qualitative approach is well suited to explaining why similar intentions lead to different actions and why formal provision may not become usable opportunity. Student focus groups can reveal negotiated norms, disagreements about responsibility, and collective efficacy, while faculty interviews can illuminate curriculum, modeling, resource constraints, and organizational inconsistency. Comparing the two stakeholder groups also exposes mismatches between institutional assumptions and lived experience, including contradictions and minority perspectives that formal models can overlook.

Accordingly, this multi-site qualitative study used student focus groups and semi-structured faculty interviews to explain how environmental intention becomes, or fails to become, private and public campus action. The institutionally situated extended TPB served as a sensitizing and interpretive framework rather than a fixed coding template. The study’s objective was to identify the personal, social, pedagogical, and institutional mechanisms that condition action and to derive evidence-linked implications for curriculum, campus operations, participation, and governance.

## Literature review

Prior research provides robust evidence that attitudes, norms, perceived control, knowledge, nature connection, personal norms, and efficacy are associated with pro-environmental behavior (Geiger et al., 2019; Nisbet et al., 2009; Otto and Pensini, 2017; Silvi and Padilla, 2021). However, three limitations remain. First, many studies model additional variables as parallel predictors without clarifying their conceptual position relative to the TPB. Second, private and public behaviors are often combined even though they involve different levels of visibility, coordination, and institutional dependence. Third, cross-sectional models reveal associations but not how students and faculty interpret unreliable systems, contradictory norms, or unresponsive participation structures. The review therefore develops one coherent perspective—an institutionally situated extended TPB—and identifies the qualitative gaps addressed by the study.

### Campus sustainability as a behavioral context

Campus sustainability encompasses curriculum, teaching, governance, operations, student life, community engagement, and organizational culture (Dagiliūtė et al., 2018; Findler et al., 2019). Universities are not merely settings in which environmental attitudes are expressed; they are organizations that allocate authority, create defaults, maintain infrastructure, and signal priorities. Recent studies in higher education likewise show that sustainability education is more consequential when institutional support and operational practice are aligned with learning opportunities (Chen and Erfanian, 2026; Qi et al., 2025).

Students encounter institutional messages through courses, recycling and energy systems, transport, food services, procurement, residence rules, student organizations, and decision-making opportunities. Alignment among curriculum, infrastructure, faculty behavior, and policy can strengthen credibility; inconsistency can make initiatives appear symbolic. Work on educational environmental management similarly emphasizes that training, institutional organization, participation, and the socially constructed meaning of waste-management systems shape whether environmental ideas become practice (Vilchis Pérez et al., 2021, 2023, 2026). Campus behavior must therefore be understood as both psychologically motivated and organizationally enabled.

### Private and public pro-environmental behavior

Pro-environmental behavior is not a single outcome. Private campus behavior includes conserving water and electricity, reducing waste, recycling, using reusable products, and making sustainable transport or purchasing choices (Gkargkavouzi et al., 2019; Larson et al., 2015; Markle, 2013; Mateer et al., 2022). Public behavior includes joining campaigns, encouraging peers, sharing environmental information, participating in organizations, and advocating for institutional initiatives (Markle, 2013; Stern et al., 1999). The distinction is analytical rather than moral: personally performed routines can depend heavily on institutional systems, while visible participation can be episodic or symbolic.

The two domains require partly different mechanisms. Private behavior is often organized through habits, convenience, cost, access, and personal control. Public behavior additionally requires legitimacy, coordination, organizational continuity, collective efficacy, and confidence that authorities will respond (Bandura, 2000; Helferich et al., 2023). Students may therefore be consistent in one domain and inconsistent in the other. Treating them separately prevents one type of action from serving as an invalid proxy for the other and permits intervention strategies to be matched to distinct barriers.

### Theoretical perspective: an institutionally situated extended TPB

The TPB proposes that behavior follows most directly from intention and perceived behavioral control, and that intention reflects behavioral attitudes, subjective norms, and perceived control (Ajzen, 1991, 2011, 2020). Attitude is behavior-specific evaluation, not simply general concern for the environment. Subjective norm concerns perceived social expectations, while perceived behavioral control concerns the person’s appraisal of capability and opportunity. Actual control—the resources and constraints that exist in the situation—determines whether a well-formed intention can be enacted. This distinction allows a student to feel personally capable yet remain constrained by a broken, inaccessible, unaffordable, or distrusted campus system.

Agency in this perspective is situated. Deliberate intention matters, but habits, defaults, health and safety requirements, time pressure, and institutional rules shape the range of feasible choices. Recent campus research in China supports the continued relevance of TPB pathways while also showing the importance of institutional context and extended antecedents (Chen and Erfanian, 2026). The present study does not test a larger causal model; it examines how participants connect these constructs and conditions in accounts of action and non-action.

### Knowledge, connectedness, and attitudes

Environmental knowledge includes system knowledge, procedural knowledge, and effectiveness knowledge (Frick et al., 2004; Kaiser and Fuhrer, 2003). Knowledge can inform attitudes by clarifying consequences and can strengthen perceived control by clarifying how to act. Yet generic awareness may be behaviorally weak when local procedures are unknown, an action channel is absent, or the institution does not reveal whether a practice is effective. Classroom dialogue and participatory inquiry can make environmental meanings more concrete, but knowledge remains only one antecedent of action (Cardona Castaño et al., 2021; van de Wetering et al., 2022).

Connectedness to nature is a cognitive, emotional, and experiential identification with the natural world and is associated with environmental concern and behavior (Barrera-Hernández et al., 2020; Mayer and Frantz, 2004; Tam, 2013; United Nations, 2015; Whitburn et al., 2020). Within the present framework, it is a distal source of meaning rather than a necessary condition. Campus action may also be evaluated through health, efficiency, fairness, professional responsibility, civic duty, or cost. Multiple motivational routes are therefore expected.

Pro-environmental attitude requires more theoretical precision than a favorable view of sustainability in general. In the TPB, the relevant attitude is an evaluation of a specific behavior under particular conditions: for example, whether using a reusable container, sorting waste, or joining a campaign is useful, pleasant, fair, safe, and worth the effort. Instrumental beliefs about effectiveness can coexist with experiential concerns about inconvenience and moral beliefs about responsibility. Consequently, a student may endorse environmental protection yet evaluate a particular campaign negatively because it appears costly, performative, or technically weak. Feedback, visible outcomes, and institutional credibility can therefore change the behavioral beliefs from which attitudes are formed (Ajzen, 2020; Bamberg and Möser, 2007).

### Social, moral, and collective processes

Subjective norms concern perceived expectations from peers, roommates, faculty, families, student organizations, and administrators (Ajzen, 1991; La Barbera and Ajzen, 2020). Official sustainability messages may have little influence when responsible behavior is uncommon, whereas visible peer and faculty participation can normalize action. Focus groups can reveal differences between formal institutional expectations and authentic peer approval.

Personal norms refer to an internalized moral obligation to act and are central to norm-activation and value-belief-norm accounts (Schwartz, 1977; Stern et al., 1999). Moral responsibility may arise from knowledge, values, nature connection, education, or prior participation. However, emphasizing individual responsibility can create guilt or blame when students lack influence over procurement, heating, transport, or waste systems. Qualitative inquiry should therefore examine how students and faculty divide responsibility among individuals, universities, governments, and corporations.

Perceived behavioral control is the participant’s appraisal of whether action is within reach; it can reflect confidence, knowledge, time, affordability, convenience, facilities, safety, and participation opportunities (Ajzen, 1991, 2020). Institutional reliability is conceptually different: it is the experienced condition of systems, procedures, information, maintenance, and responsiveness that supplies or removes actual opportunity. Reliability may therefore act as a contextual antecedent of perceived control and as a moderator of the intention-behavior relationship. This distinction avoids treating broken infrastructure as a deficit in personal competence.

Collective efficacy is the shared belief that a group can organize and achieve desired outcomes (Bandura, 2000). It is particularly relevant to public behavior because collective action requires peer coordination and an institutional audience capable of responding. Successful projects, access to data and decision-makers, resources, and visible feedback can strengthen efficacy; symbolic consultation and administrative silence can weaken it. Participatory approaches outside universities also show that co-produced knowledge becomes consequential only when collective interpretation is connected to viable decision pathways (Cardona Castaño et al., 2025).

### Faculty roles and need for qualitative inquiry

Faculty members can influence knowledge, behavioral beliefs, norms, and opportunities through curriculum, assessment, research, service learning, fieldwork, mentoring, and personal modeling. Training may broaden teachers’ environmental representations and their capacity to connect disciplinary learning with collective practice (Vilchis Pérez et al., 2023). Yet faculty action is also constrained by workload, disciplinary boundaries, incentives, data access, and contradictions between teaching and operations.

The theoretical contribution sought here is therefore not an ever-larger list of predictors. It is a relational account of how distal motivations become behavior-specific attitudes and intentions, how institutional reliability shapes perceived and actual control, and how collective efficacy links public intention to consequential participation. Planned change research adds an implementation question: whether universities align leadership, resources, communication, curriculum, and operational feedback around an explicit change pathway (Bookbinder et al., 2024; Rieg et al., 2021). Existing literature rarely examines these relationships through paired student and faculty accounts.

The present study therefore used student focus groups and individual faculty interviews to analyze convergence, divergence, and institutional blind spots. Deductive concepts from the selected framework organized initial attention, while inductive analysis remained open to habit, environmental fatigue, distrust, perceived hypocrisy, workload, economic pressure, symbolic participation, disciplinary culture, fairness, and student voice. This design addressed the gap between formal behavioral relationships and the lived conditions through which they acquire meaning.

## Method

### Research design, context, and questions

This qualitative study examined how university students and faculty understood the movement from environmental intention to private and public campus action. Qualitative description supported practice-oriented accounts that remained close to participants’ language and experience (Sandelowski, 2000). The institutionally situated extended TPB was used as a sensitizing and interpretive framework: it informed the protocols and initial codes, but did not predetermine final themes or require every passage to fit a theoretical category.

Four student focus groups were used because campus environmental behavior is socially embedded and group interaction can reveal shared norms, disagreement, and collective experience (Krueger and Casey, 2015). Ten individual faculty interviews provided privacy for discussing teaching, institutional inconsistency, resource constraints, and university policy. The study involved 10 universities in eastern China. Institutional classifications grouped humanities, social sciences, education, business, and languages as arts, and natural sciences, engineering, computing, medicine, health sciences, and agriculture as sciences.

The general objective was to explain how personal agency, social and moral processes, faculty practice, and institutional conditions jointly shape the translation of environmental intention into private and public campus behavior. The study addressed three research questions (RQs):

*RQ1.* How do university students describe the personal, social, and institutional factors that facilitate or constrain their private and public campus pro-environmental behavior?

*RQ2.* How do faculty members understand their pedagogical roles and the wider institutional conditions influencing students’ private and public campus pro-environmental behavior?

*RQ3.* How do student and faculty perspectives converge or diverge regarding the translation of environmental intention into private and public campus action?

### Ethics statement

Ethical approval was obtained from the relevant institutional ethics committee. Participation was voluntary and unrelated to grades, supervision, services, employment, or evaluation. Consent covered participation, recording, transcription, analysis, and anonymized quotation. Participants could decline questions or withdraw before the anonymization deadline.

Focus-group participants were informed that researchers could protect records but could not guarantee confidentiality among group members. Faculty quotations were carefully de-identified because discipline, rank, role, and institution could reveal identity; distinctive details were generalized or omitted.

### Participants and sampling

Thirty participants were recruited purposively: 20 students and 10 faculty members. Four five-person student groups were homogeneous by educational level and discipline: undergraduate arts, undergraduate sciences, graduate arts, and graduate sciences. This arrangement reduced status differences while allowing cautious exploration of educational and disciplinary contrasts.

Eligible students were at least 18, enrolled in an undergraduate or graduate program, had attended the institution for at least one semester, had sufficient campus experience, and consented to participation and recording. Variation was sought in gender, year, residence, environmental-activity experience, and self-described engagement.

The faculty sample comprised five arts and five science participants. Eligibility required current university teaching, at least 1 year of experience, membership in an arts or science discipline, capacity to discuss student learning or campus sustainability, and informed consent. Environmental specialization was not required. Variation was sought in gender, rank, experience, discipline, sustainability-related teaching, campus involvement, and teaching level.

Sample adequacy was assessed through information power rather than a numerical saturation rule (Malterud et al., 2016). The study had a narrow aim, specific participant groups with direct campus experience, theory-informed protocols, approximately 60-min focus groups and 50-min interviews, and an analytic strategy that compared two complementary stakeholder datasets. Across the four groups and 10 interviews, the core explanatory mechanisms—situated friction, system reliability, trust, differentiated responsibility, and institutional response—recurred while negative cases and disciplinary emphases added variation. These features provided sufficient conceptual depth to answer the descriptive and interpretive research questions.

This judgment does not imply that thematic saturation or representativeness was achieved. Because each educational-level-by-discipline category contained only one focus group, the analysis could identify contextual contrasts but could not establish stable subgroup differences. The claims are therefore mechanism-oriented and bounded to the information supplied by these participants.

### Interview protocols and data collection

Separate semi-structured protocols were developed for students and faculty using systematic interview-guide development principles (Kallio et al., 2016). Student discussions addressed private and public behavior, action and non-action, knowledge, connectedness to nature, attitudes, social and personal norms, perceived control, intention–behavior gaps, collective efficacy, and interventions. Faculty interviews examined student behavior, curricular influences, faculty modeling, institutional norms, infrastructure, participation opportunities, organizational barriers, and feasible pedagogical and policy changes.

Student focus groups lasted approximately 60 min and were conducted through Tencent Meeting by a trained moderator with no teaching or assessment relationship to participants. A second researcher recorded interactional notes. After students first defined campus pro-environmental behavior, the moderator introduced the distinction between private practices such as conserving resources, recycling, and reducing disposable use; and public actions, including campaigns, peer encouragement, information sharing, and support for university initiatives. Probes invited examples, difficulties, social influences, disagreement, and group differences. Notes documented turn-taking, agreement, disagreement, hesitation, dominant voices, and sensitive topics.

Faculty interviews lasted approximately 50 min and were conducted individually online. Questions addressed teaching background, observations of private and public behavior, disciplinary differences, knowledge–action relationships, nature connection, faculty influence, institutional consistency, material barriers, collective efficacy, student participation, interventions, unintended consequences, and interpretations of how intention becomes action. Interviewers requested concrete examples and alternative explanations.

Participants received study information, provided electronic consent, and completed brief demographic forms. Sessions were audio-recorded with permission, and field-note summaries documented major issues, interactions, preliminary interpretations, and follow-up questions. Interviews were conducted and transcribed verbatim in Mandarin Chinese. Identifying details were removed. Analysis used the Chinese transcripts; selected quotations were later translated by one bilingual researcher and checked independently by another.

### Status of co-design in the study

No co-design workshop, living-laboratory intervention, or collaborative implementation activity was conducted as part of data collection. Co-design emerged inductively when participants described preferred future arrangements in which students, faculty, and operational staff would define problems, examine data, test changes, and receive a decision response. It is therefore reported as a participant-generated conceptual and practical proposal, not as a tested method or outcome of this study. The analysis examined the proposal’s perceived value, necessary conditions, and anticipated risks; it cannot establish its effectiveness.

### Data analysis

Hybrid deductive-inductive framework analysis enabled systematic comparison across theory-informed stakeholder groups while permitting emergent explanation (Gale et al., 2013). Analysis proceeded through familiarization, initial coding, development of an analytic framework, indexing, matrix charting, category development, and interpretive mapping. Researchers repeatedly read the Mandarin transcripts, checked selected passages against recordings, and wrote memos on patterns, contradictions, emotional emphasis, and interactional context. Focus-group analysis attended to agreement, challenge, hesitation, dominant voices, and collectively negotiated norms.

Two researchers independently coded one focus-group transcript and two faculty interviews to establish a shared analytic starting point. Deductive codes represented knowledge, connectedness to nature, behavioral attitudes, subjective and personal norms, perceived behavioral control, intention, collective efficacy, and private and public behavior. Inductive codes included habit, convenience, distrust, environmental fatigue, symbolic action, academic pressure, cost, institutional inconsistency, unfairness, feedback, identity, digital participation, and administrative responsiveness. The lead analyst then coded the remaining dataset, while the second analyst reviewed a meaningful proportion of coded material, matrix summaries, negative cases, and candidate themes.

The analysts developed a provisional codebook containing definitions, inclusion and exclusion boundaries, and examples. Disagreements were resolved through contextual rereading and conceptual clarification rather than by mechanically selecting one code. The framework was revised when data did not fit the initial categories. NVivo supported storage, retrieval, and comparison; interpretation remained the researchers’ responsibility.

The transition from codes to themes involved four explicit steps. First, related codes were clustered into provisional categories, such as situational friction, system usability, system trust, equitable access, credible participation, and institutional consistency. Second, student and faculty data were charted separately in case-by-category matrices. Third, candidate themes were tested against full transcripts, divergent cases, and the distinction between private and public behavior. Fourth, themes were mapped back to the theoretical framework to determine whether they elaborated a construct, specified a contextual antecedent, or identified a relationship absent from the initial model. This process generated six explanatory themes rather than a frequency-ranked list of topics.

Triangulation operated at three levels. Data-source triangulation compared student and faculty accounts; analyst triangulation compared independent coding, review, and theme decisions; and theoretical triangulation compared deductive TPB concepts with inductive institutional explanations. Convergence strengthened an interpretation, divergence was retained as an analytic finding, and negative cases were used to revise overgeneralized claims. Theory was thus integrated after and during analysis, but empirical passages were not forced into predetermined constructs.

### Methodological boundaries

The design had four anticipated boundaries. Online interviews limited observation of material settings and nonverbal interaction; self-reports were vulnerable to recall and social desirability; theory-informed questions may have made some constructs more salient; and one focus group per educational-level-by-discipline category precluded robust subgroup comparison. The study also lacked behavioral audits, institutional records, and a co-design intervention. These constraints were addressed through neutral probes, interactional notes, negative-case analysis, cautious subgroup language, and explicit separation of participant proposals from tested outcomes.

### Trustworthiness and reflexivity

Trustworthiness addressed credibility, dependability, confirmability, and transferability (Lincoln and Guba, 1985). Credibility was strengthened through two stakeholder groups, purposive variation, open-ended probing, private–public comparisons, and negative-case analysis. Dependability was supported by an audit trail of recruitment, protocol versions, field notes, codebook changes, memos, and meeting records. Confirmability was enhanced through collaborative analysis, reflexive documentation, contradictory evidence, and quotations representing common and minority perspectives. Transferability was supported through contextual description. Reporting was reviewed against the Consolidated Criteria for Reporting Qualitative Research (Tong et al., 2007).

Researchers prepared positionality statements addressing disciplinary background, institutional relationships, sustainability involvement, assumptions about student behavior, views of individual and institutional responsibility, and expectations arising from the literature and sensitizing framework. Reflexive journals documented possible encouragement of socially desirable responses or overreliance on predetermined theoretical categories. Neutral probes invited skepticism, resistance, non-participation, and perceived disadvantages.

## Results

### Overview

The dataset comprised 45-person student focus groups and 10 faculty interviews divided equally between arts and sciences. Hybrid deductive-inductive analysis generated six themes. Each theme is presented first in participants’ terms and then interpreted through the selected framework. The analysis retained intention, attitudes, perceived control, norms, knowledge, and collective efficacy while identifying institutional reliability, equity, disciplinary meaning, and student voice as conditions that organize these constructs in campus life.

Private and public behaviors were related but distinct. Private action was routine and embedded in habits, defaults, and infrastructure; public action additionally required social coordination, visibility, and confidence that participation could produce results.

### Theme 1: intention was necessary, but situated conditions determined action

Students distinguished broad environmental support from specific choices. Intention was morally agreeable but insufficient when action involved convenience, workload, habit, health, safety, and social expectations. As one graduate arts student stated, “*Intention is general, while behavior is situated*” (GA1). Faculty agreed that intention mattered only when expectations were clear and options practical.

Private behavior was most stable when it became habitual or effortless. Students consistently used reusable bottles, switched off lights, or took public transport when these actions fitted existing routines. “*When the environmental option becomes the easiest option, I do it without thinking. When it requires planning, I am less consistent*” (UA5). Examination periods, clinical schedules, employment, heat, pollution, and family obligations shifted decisions toward convenience. Medical participants emphasized that disposables or alternative transport could be justified by infection control or safety. Non-action therefore did not necessarily indicate weak concern.

Motives were often mixed. Participants conserved resources to save money, used public transport for convenience, or joined activities for friendship, learning, or recognition. Environmental identity was only one motive.

Private and public behaviors also involved different demands. Private behavior fitted routines and involved relatively low social risk, whereas public action required time, confidence, information, coordination, and visibility. “*Private behavior fits routine; public behavior requires identity and time*” (UA5). Some students maintained strong private habits but avoided campaigns they viewed as inefficient, while others joined visible activities despite inconsistent daily practices. Participants therefore treated the two domains as distinct activity systems rather than levels of the same moral commitment.

Theoretically, these accounts described situated agency. Participants could form intentions and make reflective choices, but agency was enacted within habits, defaults, competing obligations, and actual opportunities. The data therefore did not negate the TPB; they specified why intention had variable behavioral force. Private action often became automatic through supportive routines, whereas public action remained more deliberative and socially exposed.

### Theme 2: perceived control was experienced as institutional reliability

Students described control less as confidence than as whether campus systems worked. Relevant conditions included refill stations, clear bin labels, reliable recycling, building controls, safe laboratory procedures, public transport, reusable-product availability, and affordable purchasing options.

“*I knew it was not ideal, but convenience won. I do not think that is a moral failure; it is an infrastructure failure*” (UA4). Overflowing bins, ignored no-cutlery requests, delayed repairs, and inconvenient reusable systems showed that formal availability did not ensure usable opportunity.

Trust and information quality were also part of control. Students disengaged from sorting when cleaners appeared to recombine waste or when downstream processing was unclear. “*Trust is part of control. If I do not trust the waste system, I do not feel my behavior is effective even if a bin is available*” (UA2). Faculty likewise emphasized operational control, data quality, accessible communication, and clear procedures.

Defaults, maintenance, affordability, safety, and equity defined the limits of feasible action. “*The default setting is powerful. If the sustainable option is not the default, intention loses*” (FS2). Participants also warned that interventions should not assume that all students could carry containers, walk long distances, pay more, attend after-hours events, or disregard health and disability needs. Control was therefore material and distributive: participants asked both whether an option existed and who could realistically use it.

Across accounts, three broader intention-constraint patterns appeared: formal availability without practical usability; usability without trust that the action would be completed as intended; and usable, trustworthy options that remained inequitable because of cost, time, mobility, health, or safety. Perceived behavioral control refers to participants’ appraisal of these opportunities. Institutional reliability refers to the contextual systems that generated that appraisal. The distinction explains why strong intention and personal competence could coexist with non-action.

### Theme 3: collective efficacy required credible participation and visible response

Collective efficacy depended on interaction with decision-makers and evidence that participation could affect outcomes. Students who submitted proposals without receiving feedback became less willing to participate again. “*Repeated non-response teaches students that participation is ineffective*” (GA2).

Faculty agreed that collective efficacy was relational and institution-dependent. Students described consultation without authority as symbolic and requested clear procedures identifying who reviewed proposals, when decisions would be made, and why suggestions were accepted or rejected.

Successful public action left a visible trace. Clothes swaps, energy competitions using actual consumption data, compost pilots, biodiversity surveys, and course-based interventions were considered credible because outcomes were observable. “*Students need to see that their effort changed something beyond a photograph*” (FA1). Small wins, such as changing one canteen practice or improving one building system, were viewed as foundations for larger initiatives. Participants accepted that proposals might fail but expected transparent evaluation.

Continuity also required budgets, technical support, data access, leadership succession, meeting space, and legitimate sponsorship. “*Groups need resources, competence, and access to decision-makers. Motivation alone is insufficient*” (FA4). Faculty mentorship could sustain projects, but excessive administrative control risked turning student initiatives into publicity. Participants preferred student ownership supported by clear institutional commitments.

Within the framework, peer confidence alone was insufficient for collective efficacy. Efficacy was relational: students judged group capability through the university’s willingness to provide decision access, resources, continuity, and feedback. Institutional responsiveness therefore functioned as an experiential source of collective efficacy for public behavior.

### Theme 4: knowledge became actionable when it was local, practical, and meaningful

General awareness of climate change, plastic waste, and conservation was widespread. The more important gap involved local procedural and effectiveness knowledge: what belonged in each bin, where materials went, who controlled procurement, and which actions produced meaningful reductions. “*What is missing is local knowledge: where the waste goes, which bins accept what, and whether the university actually recycles*” (UA5).

Arts participants called this policy literacy; science participants emphasized system, procedural, data, and effectiveness knowledge. Specialist knowledge prevented technically weak or unsafe actions, although it could also increase doubt toward low-impact campaigns.

More information did not automatically promote action. Repetitive messages caused fatigue, while awareness of structural problems generated frustration when students lacked authority. Faculty described knowledge-to-action as situated practice involving investigation, intervention, feedback, reflection, and revision. Knowledge became action competence rather than factual awareness alone.

Environmental meaning followed multiple pathways. Nature connection motivated some participants through fieldwork, wetlands, campus gardens, old trees, and place-based learning. Others were motivated by efficiency, health, justice, professional responsibility, civic duty, or cost. “*I do not have a strong nature identity, but I dislike waste because it is inefficient*” (US3). Arts participants more often emphasized meaning, identity, communication, and governance; science participants more often emphasized data, safety, system design, and operational effectiveness. These were differences of emphasis rather than fixed disciplinary divisions.

These findings positioned knowledge and nature connection as distal rather than deterministic influences. Local effectiveness knowledge shaped beliefs about whether an action was worthwhile, thereby informing attitudes; procedural knowledge shaped perceived control. Institutions remained central because they controlled access to the local information, data, and action channels that allowed knowledge to acquire behavioral relevance.

### Theme 5: institutional consistency shaped norms, trust, and responsibility

Participants distinguished formal messages from norms communicated through operations. Campaigns lost credibility when events generated disposable waste, courses required unnecessary printing, procurement favored wasteful options, or departments failed to coordinate. “*We promote paper reduction and then require multiple printed forms. Students notice contradictions quickly*” (FA1).

Students often perceived an informal norm of avoiding inconvenience that overrode official sustainability claims. Faculty interpreted this as fragmented governance across communication, budgeting, facilities, procurement, health, and academic units. Such fragmentation signaled what the university genuinely prioritized.

Moralizing communication also provoked resistance. Students accepted responsibility for choices under their control but rejected blame for structural failures. “*Responsibility should match control. If I cannot change the heating system or packaging contracts, it is unfair to blame me*” (UA4). Faculty similarly favored invitation, reflection, and achievable roles over guilt or public performance.

The preferred model was differentiated shared responsibility. Students were responsible for routine choices; faculty for curriculum, modeling, and mentorship; operational units for functional systems; and senior leaders for budgets, procurement, governance, and responses to proposals. “*Responsibility should be distributed according to authority and capacity*” (GA3). Health, disability, finances, residence, and professional safety requirements shaped capacity, making fairness integral to sustainability.

Institutional consistency consequently operated through several TPB pathways at once: it supplied behavioral information, communicated descriptive and injunctive norms, affected trust and perceived control, and altered evaluations of whether action was worthwhile. From a planned-change perspective, contradictory messages signaled the absence of a shared change pathway across curriculum, procurement, facilities, health, and governance.

### Theme 6: co-designed living laboratories emerged as a bounded proposal for institutional learning

Faculty did not define their role primarily as providing more information. They emphasized assignments, standards, modeling, and authentic institutional problems. Arts faculty proposed place-based writing, debates, oral histories, reflective inquiry, and institutional ethnography. Science faculty proposed monitoring, green-laboratory audits, design cycles, data projects, and health-impact assessment. “*Students should investigate a real problem, try an intervention, observe the result, and revise*” (FA2). These suggestions described co-design as shared problem definition and iterative inquiry, not merely student consultation after decisions had been made.

Both groups envisioned a living laboratory linking courses with facilities, procurement, health services, student affairs, and sustainability units. Their proposed sequence included a bounded campus problem, shared definition of the question, access to appropriate data, co-development of feasible options, a small-scale test, transparent review by a named decision-maker, and evaluation of outcomes. “*Universities should treat students as co-researchers and co-designers of sustainability systems*” (GS1). This proposal linked individual agency to collective efficacy by giving participants a credible route from knowledge and intention to institutional action.

Participants nevertheless identified limits. Co-design could reproduce unequal voice if confident students or favored disciplines dominated; operational partners might restrict data; projects could add workload, create privacy risks, or end as publicity; and technically successful pilots might fail to scale. Dashboards were acceptable only when data were interpretable and protected against surveillance, gaming, and unequal pressure. Co-design therefore required facilitation, accessibility, explicit decision boundaries, ethical data governance, and recognition of students’ time.

Long-term institutional integration was preferred to occasional campaigns. Participants recommended course-operations partnerships, central coordination, public response timelines, small grants, succession plans, and evaluation beyond project launch. “*A pilot that never moves into policy becomes symbolic*” (GS1). Because no co-design activity was implemented in this research, the finding establishes perceived desirability and design conditions, not intervention effectiveness.

### Student–faculty convergence and divergence

As Table 1 shows, students and faculty converged on four claims: (a) intention alone did not explain action; (b) perceived control depended partly on system reliability and trust; (c) public behavior required voice, resources, and visible response; and (d) individual responsibility required institutional accountability. Faculty translated broken facilities, confusing bins, ignored proposals, and inconsistent rules into poor default design, fragmented governance, weak feedback, and curriculum-operations separation. The integrated interpretation was that institutional conditions shape actual control, participants’ perceptions of control, and the collective efficacy needed for public action.

**Table 1:** Convergence and divergence of student and faculty perspectives.

Analytic issue	Student perspective	Faculty perspective	Theory-informed integrated interpretation
Intention–action gap	Habit, workload, convenience, health, cost, and distrust interrupted action	Defaults, fragmented systems, and weak feedback shaped implementation	Situated agency: intention was conditional on friction and actual opportunity
Perceived control	Working facilities, clear procedures, affordable options, and trustworthy systems	Operational control, interface design, data access, accessibility, and safety	Perceived control was an appraisal; institutional reliability was a contextual antecedent and source of actual control
Public behavior	Voice, peer support, time, legitimacy, and evidence that leaders listened	Resources, sponsorship, continuity, competence, and implementation authority	Collective efficacy was relational and depended on credible institutional response
Knowledge and motivation	Local knowledge and multiple motives mattered more than generic awareness	Practice, disciplinary relevance, feedback, and reflection converted knowledge into action	Knowledge and nature connection informed attitudes and control only when linked to local action channels
Responsibility	Students rejected blame for structural failures	Faculty called for alignment across curriculum, operations, and governance	Normative accountability was shared but differentiated according to authority and capacity
Preferred interventions	Reliable services, transparent results, long-term projects, and co-design	Living laboratories, partnerships, response channels, and evaluation	Planned change required bounded co-design, operational partnership, feedback, evaluation, and continuity

Also, as shown in Table 1, differences were primarily matters of emphasis. Student activists saw public events as opportunities for norm formation, whereas doubtful students prioritized measurable system change. Arts participants more often emphasized identity, voice, moralizing, and trust; science participants more often emphasized data, safety, and system performance. Faculty stressed educational scaffolding and system design, while students emphasized whether prior institutional action had earned trust. Nature connection sustained some participants; health, efficiency, justice, professional ethics, and civic responsibility motivated others.

### Summary by research question

For RQ1, students described private and public behavior as products of interacting personal, social, and institutional conditions. Habit, workload, cost, health, and mixed motives mattered, but so did infrastructure, maintenance, defaults, information quality, trust, and voice. Public behavior additionally required confidence that participation was legitimate and consequential.

For RQ2, faculty understood their role as connecting knowledge to action through modeling, assignments, standards, feedback, and real campus projects. They identified fragmented governance, weak institutional response, limited data access, and separation between curriculum and operations as major barriers.

For RQ3, the groups largely converged on operational reliability, institutional credibility, visible feedback, differentiated responsibility, and meaningful participation. Students emphasized lived inconvenience, authenticity, and trust; faculty framed similar issues through pedagogy, governance, and system design. Together, the findings show that institutional trust and responsiveness condition perceived control and collective efficacy, while the intention–action relationship is shaped by friction, equity, and alignment between university messages and practice.

## Discussion of findings

### Situated agency and the conditional force of intention

The study supports the TPB proposition that intention is a proximal readiness to act while behavior also depends on perceived and actual control (Ajzen, 1991, 2020). Its added value is to conceptualize the intention-behavior gap as situated agency rather than personal inconsistency. Workload, convenience, health, safety, and habits altered the feasible choice set, while institutional systems determined whether intended action could be completed. Agency was therefore neither absent nor unlimited: it was exercised through the opportunities that campus organization made credible.

This interpretation prevents a moralizing inference from non-action to weak concern. A student who used disposables for infection control or stopped sorting after observing recombined waste was responding to changed consequences and expected effectiveness. These accounts show how behavior-specific attitudes and perceived control can be revised by experience even when general environmental concern remains stable.

Campus pro-environmental behavior is thus institutionally situated. Universities govern residences, laboratories, food services, transport, procurement, and participation structures; these arrangements shape actual control and supply evidence from which behavioral beliefs are formed. Making sustainable options dependable, affordable, accessible, and routine is not external to behavioral explanation. It is part of the causal context through which intention acquires or loses behavioral force.

### Private and public behavior as distinct systems

The private-public distinction reveals two related but non-equivalent action systems. Private behavior often relied on habit, low-friction defaults, and personal routines. Public behavior added identity exposure, coordination, organizational continuity, and uncertainty about institutional response. This extends multidimensional accounts of environmentally significant behavior (Stern, 2000) by specifying why public intention requires a collective and governance mechanism beyond individual perceived control.

Neither domain was morally superior or an adequate proxy for the other. Strong private routines could coexist with distrust of campaigns, while visible participation could coexist with inconsistent daily practice. The implication is theoretical and practical: private action is more directly explained by individual intention interacting with reliable defaults, whereas public action requires collective efficacy, legitimate participation, and an institution capable of responding.

Private behavior may respond most directly to dependable infrastructure, maintenance, defaults, reminders, and resource access. Public behavior requires trusted invitations, clear roles, protected time, continuity, access to decision-makers, and evidence that participation matters. One behavioral domain should not be used as a proxy for the other.

### Perceived control as institutional reliability

Perceived behavioral control concerns a person’s judgment of capability and opportunity (Ajzen, 1991, 2020). Institutional reliability is analytically prior to, but not identical with, that judgment. Working facilities, clear procedures, maintenance, credible information, affordability, safety, accessibility, and dependable follow-through are contextual properties that participants use when forming control beliefs.

The theoretical contribution is a two-level distinction. At the individual level, perceived control captures whether action feels feasible. At the contextual level, institutional reliability captures whether the university actually supplies and completes the action pathway. Bins can exist without being usable; a system can be usable without being trusted; and a trusted option can remain inequitable. These three patterns explain apparent overlap in the results while preserving conceptual boundaries.

Control was also distributive. Formal provision that requires extra money, time, mobility, storage, or after-hours attendance creates unequal actual opportunity. Equity is therefore not an additional attitude variable but a property of the control environment. Behavioral research and campus evaluation should ask who can use an option without disproportionate cost or risk.

### Collective efficacy and credible participation

Collective efficacy was not simply confidence that students could cooperate. It was learned relationally through whether collective effort received resources, access, feedback, and implementation. This elaborates Bandura’s (2000) group-capability concept by identifying institutional responsiveness as an experiential source of efficacy in hierarchical organizations.

Repeated consultation without response produced a negative mastery history: it taught participants that organized effort was inconsequential. Small wins and transparent decisions had the opposite effect. Public participation should therefore be understood as a reciprocal system in which student coordination and institutional response jointly produce or erode collective efficacy.

Public participation should therefore be designed as co-governance rather than audience participation. Students need clarity about who reviews proposals, what is open to influence, when responses will be provided, and how implementation will be evaluated. Universities need not accept every proposal, but unexplained silence damages trust.

### Knowledge, nature connection, and multiple motivations

Participants generally possessed broad environmental awareness. The more important deficit involved local procedural, policy, and effectiveness knowledge: what actions were possible, where materials went, who controlled systems, and whether interventions worked. This aligns with distinctions among system, action-related, and effectiveness knowledge (Frick et al., 2004; Kaiser and Fuhrer, 2003).

Knowledge became useful when connected to an action channel. This finding challenges an information-deficit approach: additional messages did not compensate for absent authority, unclear procedures, or untrustworthy systems. Local procedural and effectiveness knowledge informed perceived control and behavioral attitudes because it clarified what could be done and whether the effort was consequential.

Connectedness to nature motivated some participants through fieldwork, green spaces, and place attachment, consistent with research linking nature connection to concern and action (Mayer and Frantz, 2004; Whitburn et al., 2020). Others were motivated by health, efficiency, justice, professional ethics, civic responsibility, or cost. Arts participants more often emphasized identity, voice, fairness, and power, while science participants emphasized measurement, safety, and system performance. These perspectives were complementary.

### Institutional consistency and differentiated responsibility

Participants interpreted university norms through operational practice as well as formal messages. Procurement, assessment, facilities, and administrative routines acted as behavioral evidence about what the institution actually valued. Institutional consistency therefore shaped subjective norms, attitudes toward specific initiatives, and trust in action pathways simultaneously.

Students accepted responsibility for choices within their control but resisted blame for structural decisions beyond their authority. Shared but differentiated responsibility resolves this tension by allocating obligations according to capacity: students enact feasible routines; faculty integrate and model sustainability; operational units maintain systems; and leaders align budgets, procurement, governance, and accountability. This preserves agency without individualizing institutional failure.

Moralizing communication should also be avoided. Shame may generate resistance, fatigue, or performative compliance. Participants preferred achievable roles, transparent evidence, and acknowledgment of constraints.

Planned change provides an implementation logic for this interpretation. A credible change pathway should name desired outcomes, identify assumptions and barriers, align responsible units, specify resources and feedback, and test whether operational changes alter control and efficacy (Bookbinder et al., 2024; Rieg et al., 2021). Campaigns without this alignment may increase awareness while leaving the action system unchanged.

### Faculty roles and living laboratories

Faculty influence operated through curriculum, assignments, standards, mentorship, feedback, and modeling. Participants’ living-laboratory proposal connected these pedagogical functions with campus operations and governance. This is consistent with work showing that campus living labs require organizational support, facilitation, continuity, and a route from experimentation to decision-making (Herth et al., 2025). It also aligns with participatory approaches that value locally situated knowledge and shared interpretation (Cardona Castaño et al., 2025).

The proposed model is bounded rather than celebratory. Operational partners would need to share appropriate data, define what is open to influence, protect privacy, respond to recommendations, and support evaluation. Facilitators would need to prevent tokenism and unequal voice, recognize student workload, and plan for succession and scaling. Because the present study did not implement co-design, these are empirically grounded design propositions, not evidence that living laboratories will necessarily change behavior.

### Theoretical and practical implications

The findings make five theoretical contributions. First, situated agency explains why intention has conditional behavioral force. Second, institutional reliability is distinguished from perceived control as a contextual source of actual and perceived opportunity. Third, collective efficacy for public behavior is produced relationally through credible institutional response. Fourth, knowledge and nature connection influence action through behavior-specific attitudes, control, and available action channels rather than as direct guarantees. Fifth, private and public behavior require partly different explanatory and intervention systems.

Practical recommendations follow directly from these mechanisms. To strengthen control, universities should publish service standards and maintain reliable recycling, refill, transport, energy, and reusable-product systems. To strengthen collective efficacy, they should define decision spaces, name responsible contacts, provide response deadlines, modest resources, and transparent reasons for decisions. To convert knowledge into competence, curricula should use local data and bounded campus problems. To support planned change, institutions should coordinate curriculum, facilities, procurement, health, student affairs, and leadership around measurable outcomes rather than campaign visibility.

The co-design proposal can be piloted through short course-operations partnerships and facilitated workshops in which students, faculty, and staff define one problem, test one feasible change, and evaluate access, environmental outcomes, trust, and participation quality. Institutional or public policy can support these pilots through sustainability reporting requirements, small-grant programs, data-governance standards, staff development, and curriculum incentives. Community partnerships may extend the approach beyond campus, but transfer should be evaluated rather than assumed.

### Limitations and future research

This study has several limitations. The sample comprised four student focus groups and 10 faculty interviews, with only one group in each educational-level-by-discipline category. Information power supported the focused interpretive aim, but it does not establish saturation, representativeness, or stable subgroup differences. Focus-group interaction may have amplified dominant positions, and volunteers may have been unusually interested in sustainability.

The cross-sectional, online, self-report design cannot establish causality and may be affected by recall, social desirability, and limited observation of nonverbal interaction. Theory-informed prompts may have foregrounded TPB concepts, although inductive coding and negative-case analysis reduced this risk. The study did not include direct observation, resource-use records, proposal outcomes, administrators or operational staff, and it did not implement the co-design or living-laboratory approach that participants proposed.

Future research should combine interviews with recycling audits, energy and transport data, participation logs, and proposal outcomes. Longitudinal designs could test whether changes in reliability precede changes in perceived control and whether responsive participation strengthens collective efficacy. Co-design and living-laboratory pilots should use comparison or interrupted time-series designs where feasible, include operational staff and decision-makers, document unequal participation and unintended burdens, and evaluate both environmental outcomes and governance quality across institutions and cultural contexts.

## Conclusion

The study’s general objective was achieved at a qualitative and interpretive level: it identified how personal agency, social and moral processes, faculty practice, and institutional conditions jointly shape the translation of environmental intention into private and public campus action. RQ1 was answered through students’ accounts of habit, friction, mixed motives, trust, access, and voice. RQ2 was answered through faculty accounts of curriculum, modeling, organizational fragmentation, and feasible institutional change. RQ3 was answered by showing convergence on reliability, responsiveness, differentiated responsibility, and meaningful participation, alongside differences in emphasis between lived trust and system design.

The answers refine the selected theoretical framework. Intention operates through situated agency; perceived behavioral control is an individual appraisal, while institutional reliability supplies the material and organizational conditions underlying that appraisal; and collective efficacy for public behavior depends partly on credible institutional response. Knowledge is most useful when local, procedural, and connected to action, while nature connection operates alongside health, efficiency, justice, professional ethics, and civic commitment.

The objective was not achieved in a causal or intervention-evaluation sense. The study cannot show that improving reliability or implementing co-design will change behavior, and the proposed living-laboratory model was not tested. These limits define the next research step rather than weaken the interpretive findings.

The broader implication is that universities and education authorities can move from awareness campaigns to coordinated action systems. The findings can inform campus sustainability policy, curriculum development, faculty and staff training workshops, student participation protocols, small-grant living-laboratory programs, and community co-design initiatives. Their value will depend on bounded decision authority, equitable access, transparent feedback, ethical data practices, and evaluation of environmental and governance outcomes.

## Data Availability

The raw data supporting the conclusions of this article will be made available by the authors, without undue reservation.
